# Diagnosis and Prediction of Neuroendocrine Liver Metastases: A Protocol of Six Systematic Reviews

**DOI:** 10.2196/resprot.2890

**Published:** 2013-12-23

**Authors:** Stephan Arigoni, Stefan Ignjatovic, Patrizia Sager, Jonas Betschart, Tobias Buerge, Elena Scherrer, Josephine Wachtl, Christoph Tschuor, Perparim Limani, Milo A Puhan, Mickael Lesurtel, Dimitri A Raptis, Stefan Breitenstein

**Affiliations:** ^1^Clinic for Visceral and Transplantation SurgeryDepartment of SurgeryUniversity Hospital ZurichZurichSwitzerland; ^2^Institute for Social andPreventive MedicineUniversity of ZurichZurichSwitzerland; ^3^Cantonal Hospital WinterthurDepartment of SurgeryClinic for Visceral and Thoracic SurgeryWinterthurSwitzerland

**Keywords:** neuroendocrine tumors (NET), liver metastases, Ki67, mitotic count, genetic signatures, tumor cells, biochemical markers, morphological imaging, functional imaging, systematic review

## Abstract

**Background:**

Patients with hepatic metastases from neuroendocrine tumors (NETs) benefit from an early diagnosis, which is crucial for the optimal therapy and management. Diagnostic procedures include morphological and functional imaging, identification of biomarkers, and biopsy.

**Objective:**

The aim of six systematic reviews discussed in this study is to assess the predictive value of Ki67 index and other biomarkers, to compare the diagnostic accuracy of morphological and functional imaging, and to define the role of biopsy in the diagnosis and prediction of neuroendocrine tumor liver metastases.

**Methods:**

An objective group of librarians will provide an electronic search strategy to examine the following databases: MEDLINE, EMBASE and The Cochrane Library (Cochrane Database of Systematic Reviews, Cochrane Central Register of Controlled Trials (CENTRAL), Database of Abstracts of Reviews of Effects). There will be no restriction concerning language and publication date. The qualitative and quantitative synthesis of the systematic review will be conducted with randomized controlled trials (RCT), prospective and retrospective comparative cohort studies, and case-control studies. Case series will be collected in a separate database and only used for descriptive purposes.

**Results:**

This study is ongoing and presents a protocol of six systematic reviews to elucidate the role of histopathological and biochemical markers, biopsies of the primary tumor and the metastases as well as morphological and functional imaging modalities for the diagnosis and prediction of neuroendocrine liver metastases.

**Conclusions:**

These systematic reviews will assess the value and accuracy of several diagnostic modalities in patients with NET liver metastases, and will provide a basis for the development of clinical practice guidelines.

**Trial Registration:**

The systematic reviews have been prospectively registered with the International Prospective Register of Systematic Reviews (PROSPERO): CRD42012002644; http://www.metaxis.com/prospero/full_doc.asp?RecordID=2644 (Archived by WebCite at http://www.webcitation.org/6LzCLd5sF), CRD42012002647; http://www.metaxis.com/prospero/full_doc.asp?RecordID=2647 (Archived by WebCite at http://www.webcitation.org/6LzCRnZnO), CRD42012002648; http://www.metaxis.com/prospero/full_doc.asp?RecordID=2648 (Archived by WebCite at http://www.webcitation.org/6LzCVeuVR), CRD42012002649; http://www.metaxis.com/prospero/full_doc.asp?RecordID=2649 (Archived by WebCite at http://www.webcitation.org/6LzCZzZWU), CRD42012002650; http://www.metaxis.com/prospero/full_doc.asp?RecordID=2650 (Archived by WebCite at http://www.webcitation.org/6LzDPhGb8), CRD42012002651; http://www.crd.york.ac.uk/PROSPERO/display_record.asp?ID=CRD42012002651#.UrMglPRDuVo (Archived by WebCite at http://www.webcitation.org/6LzClCNff).

## Introduction

### Background

#### Neuroendocrine Tumors

Neuroendocrine tumors (NETs) arise from the diffuse neuroendocrine system and therefore appear widespread over the whole body, especially in the gastrointestinal tract and the bronchopulmonary system [1,2]. NETs secreting hormones lead to a symptomatic disease. Nonsecreting NETs may occur initially asymptomatic or with delayed symptoms due to progressive increase in tumor mass [3,4]. Therefore, differences in functional behavior are the basis of a classification system categorizing functioning and nonfunctioning NETs [4]. Other reported classification systems are based on embryological origin or histopathological findings. In 2010, The World Health Organization (WHO) presented a new classification on the basis of tumor grading using histopathological criteria such as Ki67 index, mitotic count, and presence or absence of necrosis [5].

NETs is a relatively rare disease with an incidence of 1-3 per 100,000 [6,7]. The large range of reported incidence might be due to the fact that NETs often present initially asymptomatic and are often found accidentally or in autopsies [4]. Predominantly, NETs emerge sporadically (>90%) and are traditionally assigned to multiple endocrine neoplasia type 1 (MEN1), neurofibromatosis-type 1 (NF1), and Von-Hippel-Lindau syndrome [1,4]. The clinical picture of NETs spans over different effects of excessive hormone secretion such as hypergastrinemia in Zollinger-Ellison Syndrome (ZES) with hyperchlorhydria, hyperinsulinemia in insulinoma, flushing and diarrhoea in the serotoninergic carcinoid syndrome. In the case of nonsecreting NETs, symptoms present due to the adverse effects of the growing primary tumor or metastases [8].

#### Biochemical Markers

Hormones secreted from NETs can be used as specific markers for NETs. Moreover, NETs express, store, and secrete characteristic neuronal proteins such as acid glycoprotein chromogranin A (a component of the membrane neurosecretory granula), neuron-specific-enolase (NSE), and synaptophysin [3,9]. These proteins derived from neuronal structures could serve as markers and are even positive in nonfunctioning NETs [1,3]. Since more than one half of NETs are nonsecreting, these proteins play a crucial role [4]. Assessment of different biochemical markers depends on various parameters, such as threshold cut-off level, detecting method of urine, serum or plasma as well as location of the primary tumor or metastases and extension of the disease. Due to the large variety and number of evaluation parameters, it is difficult to compare the studies [10,11].

#### Histopathological Prognostic Markers

Ki67 is a monoclonal antibody, which was introduced in 1984 by Gerdes et al [11]. It detects a growth rate depending on the nuclear antigen Ki67 which is only expressed during active cell cycle phases (S, G2, and M-phase). Ki67 is completely absent during the resting phase G0. Therefore, cell proliferation is assessed by the immunohistologic presence of Ki67 positive cells per area in stained tissue blocks [11].

For various human neoplasms such as breast, lung, and solid cancers, Ki67 proliferation index has been successfully established as a predictive marker [12,13]. The higher the cell proliferation, the greater is the probability for metastases resulting in decreased patient survival. The primary location of NETs metastases is the liver [14-17]. The occurrence of hepatic metastases is a prognostic factor which strongly influences the survival of patients suffering from NET [18-20].

#### Genetic Signatures and the Presence of Circulating Tumor Cells

To stratify outcomes in patients undergoing resection of primary NET, a simple scoring system using tumor size, histological grade, nodal metastases, and resection margin status has been introduced [21]. Nevertheless, current classification systems for NETs other than positron emission tomography (PET) fail to predict the clinical course and the response to treatment [22]. The discrepancy might be explained either by an insufficient accuracy of these classification systems or an adaptive NET behavior [23]. These limitations of the pathologic classifications have led to the investigation of other predictive parameters based on genetic signatures as well as the presence of circulating tumor cells [24,25]. These novel predictive parameters have to be included in the classification systems in order to account for the biological behavior, the likelihood for developing metastases as well as the choice of treatment [25].

#### Imaging Methods

Imaging methods are used to diagnose neuroendocrine tumors (NETs) and their metastases [26]. Beside conventional morphologic imaging methods, functional imaging modalities have been introduced in order to improve accuracy in detecting NETs and liver metastases [27]. Functional imaging methods have their limitations with a great impact on a possible therapeutic strategy, where differentiation between pancreatic foci and neighbouring lymph nodes as well as exact demarcation of a suspicious focus to a liver segment is crucial [28]. Advanced techniques such as contrast-enhanced ultrasound may assist in earlier detection of hepatic metastases, and could therefore offer a wider therapeutic range either surgically, with radiofrequency thermal ablation, or with systemic chemotherapy [29].

#### Liver Biopsy

The most common site of neuroendocrine tumor (NET) metastases is the liver [30]. The presence of hepatic metastases is a strong prognostic factor for the survival of patients with NETs, regardless of the primary tumor site [31]. Histologic examination is the most sensitive diagnostic method and forms the basis for treatment decisions [32]. However, the value of the biopsy for treatment decision making involving primary NETs and their liver metastases is not well defined [33,34].

### Objective

The aim of these six systematic reviews is to assess the predictive value of Ki67 index and other biomarkers, to compare the diagnostic accuracy of morphological and functional imaging, and to define the role of biopsy in the diagnosis and prediction of neuroendocrine tumor liver metastases.

## Methods

### Systematic Reviews

Our reviews were prospectively registered at the International Prospective Register of Systematic Reviews (PROSPERO) with the following IDs: CRD42012002644, CRD42012002647, CRD42012002648, CRD42012002649, CRD42012002650, CRD42012002651.

The above six systematic reviews dealing with the diagnosis and prediction of neuroendocrine liver metastases attempt to address the following questions in Table 1.

**Table 1:** Scientific questions on diagnosis and prediction of neuroendocrine liver metastases.

Questions	Sub-questions	
**Should patients with low Ki67 index be followed up for the detection of liver metastases?**		
	In patients with a primary NET, what is the predictive value of Ki67 index, mitotic count, or tumor grading, obtained from the primary tumor, in predicting the development of liver metastases?	
**Should genetic signatures and the presence of circulating tumor cells be used in the prediction of liver metastases and to inform treatment decisions?**		
	In patients with a primary NET, what is the predictive value of genetic signatures obtained from the primary tumor, in predicting the development of liver metastases?	
	In patients with a primary NET, what is the predictive value of circulating tumor cells obtained from the primary tumor, in predicting the development of liver metastases?	
	In patients with a primary NET, should genetic signatures be used in the treatment decision (surgery, locally ablative techniques, liver-directed techniques, peptide receptor radionuclide treatment, chemotherapy, targeted therapy, and biotherapy)?	
	In patients with a primary NET, should the presence of circulating tumor cells be used in the treatment decision (surgery, locally ablative techniques, liver-directed techniques, peptide receptor radionuclide treatment, chemotherapy, targeted therapy, and biotherapy)?	
**Which biochemical markers should be used for detection and post treatment follow-up of liver metastases?**		
	In patients with a primary NET, what is the diagnostic accuracy of the available biochemical markers (eg, chromogranin A and B, Serotonin, neuron-specific-enolase (NSE), tumor specific hormones) in detecting liver metastases?	
	In patients receiving a liver resection, what is the diagnostic accuracy of the available biochemical markers (eg, chromogranin A and B, serotonin, NSE, tumor specific hormones) obtained during follow-up, in detecting recurrent disease or disease progression?	
**Which morphological imaging modality should be used to assess resectability of liver metastases with a curative intent?**		
	In patients with NET liver metastases, what is the diagnostic accuracy of different morphological imaging modalities (US, CT, MRI) in identifying liver lesions and extrahepatic disease?	
	In patients with NET liver metastases, what is the diagnostic accuracy of different morphological imaging modalities (US, CT, 3D-CT, MRI) in detecting vascular and biliary invasion, in order to assess resectability (R0/R1)?	
**Which functional imaging modality should be used to assess resectability of liver metastases with a curative intent?**		
	In patients with NET liver metastases, what is the diagnostic accuracy of different functional imaging modalities (octreoscan, DOTA-SSTR-PET/CT, F-18 FDG-PET/CT, DOPA PET, etc) in identifying liver lesions?	
	In patients with NET liver metastases, what is the diagnostic accuracy of different functional imaging modalities (octreoscan, DOTA-SSTR-PET/CT, F-18 FDG-PET/CT, DOPA PET, other) in detecting extra-hepatic disease?	
**Do we need a biopsy of both the primary and liver metastases for the treatment decision of liver metastases?**		
	In patients with a primary NET and synchronous liver metastases, what is the agreement between the biopsy of the primary and the liver metastases with regards to tumor grading?	
	In patients with metachronous liver metastases, what is the agreement between the biopsy of the primary and the liver metastases with regards to tumor grading?	
	In patients with liver metastases, what is the agreement between single vs multiple liver biopsies with regards to tumor grading?	
	In patients with NET liver metastases, do we need additional biopsies from normal parenchyma to detect micrometastases?	

**Figure 1:**
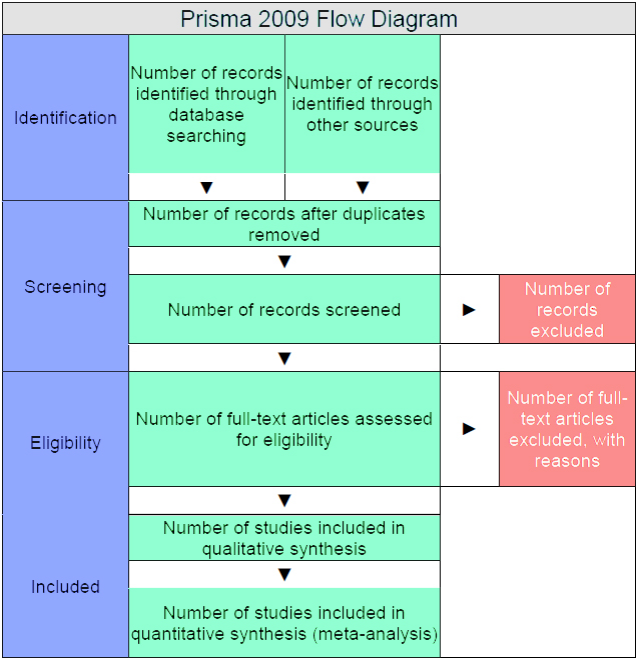
Prisma 2009 Flow Diagram.

The systematic review inclusion and exclusion criteria are listed in Tables 2-7. There were no restrictions in the literature search regarding the publication language or by publication date. The following study types were included: randomized controlled trials (RCTs), prospective and retrospective comparative cohort and case-control studies and case series (Figure 1).

**Table 2:** Eligibility criteria for review on Ki67 index.

Study characteristics	Inclusion criteria	Exclusion criteria
Participants/population	Patients with primary neuroendocrine tumors who were assessed with Ki67 index, mitotic count or tumor grading	Patients over the age of 18 years old
Tumor markers	Tumor markers (Ki67 index, mitotic count or tumor grading) must be obtained from the primary tumor	Studies that do not report the predictive value of Ki67 index, mitotic count or tumor grading
Study design	Follow-up studies for the development of liver metastases	No follow-up studies for the development of liver metastases
	Randomized controlled trials (RCTs)	
	Prospective and retrospective comparative cohort studies	Case reports
	Noncomparative cohort studies	
	Case-control studies	Reviews
	Case series	

**Table 3:** Eligibility criteria review on genetic signatures and the presence of circulating tumor cells.

Study characteristics	Inclusion criteria	Exclusion criteria
Participants/population	Patients with primary neuroendocrine tumors	Children or adolescents (under the age of 18 years old).
	Patients whose genetic signatures of the primary tumor have been tested or those who have been tested for presence of circulating tumor cells	Animal studies
		Patients with tested genetic signatures only of the metastases
	Patients with 18 years of age or older	
Test of interest	Gene expression testing of the primary tumor	Gene expression testing of the metastases
	Test for circulating tumor cells	
Reference standard	The reference standard test will be the presence or absence of liver metastases during follow-up (imaging or histopathology) by presence or absence of a genetic signature or circulating tumor cells	
Study design	Cross-sectional studies of any type	Case reports
	Cohort studies	
Reporting		Studies that do not report any predictive value

**Table 4:** Eligibility criteria for review on biochemical markers.

Study characteristic	Inclusion criteria	Exclusion criteria
Participants/population	Patients with primary neuroendocrine tumors and patients who underwent surgery for primary liver tumors with a curative intent and were followed up for the detection of potential liver metastases	Studies that do not report the assessment of resectability (second scientific question)
	Patients over the age of 18 years old	Children or adolescents (under the age of 18 years)
		Studies that do not report the diagnostic accuracy (first scientific question)
Test of interest	Tests of biochemical markers detecting liver metastases, and for the post treatment follow-up of liver metastases: 1) Chromogranin A 2) Chromogranin B 3) Serotonin 4) Tumor specific hormones (Glucose, Insulin, Proinsulin, C-Peptide, Gastrin, Glucagon, Vasoactive Intestinal Peptide, Somatostatin, Neuron Specific Enolase)	
Reference standard	The different biochemical markers Chromogranin A and B, Serotonin and tumor specific hormones will be compared	
Control	The histopathological diagnosis of the resected specimen or a tumor biopsy will be considered as the reference standard	
Study design	Randomized controlled trials (RCTs)	
	Prospective and retrospective comparative cohort studies	
	noncomparative cohort studies	
	Case-control studies	
	Case series	
Primary outcome	Diagnostic accuracy of the different biochemical markers (sensitivity and specificity)	
Secondary outcome	Additional diagnostic accuracy measures of the different biochemical markers (accuracy, positive and negative predictive values, positive and negative diagnostic likelihood ratios, etc)	

**Table 5:** Eligibility criteria for review on morphological imaging modality.

Study characteristic	Inclusion criteria	Exclusion criteria
Patient population	Patients with liver metastases from neuroendocrine tumors	Children or adolescents (under the age of 18 years)
	Patients who underwent liver transplantation or palliative liver resection or nonsurgical treatment (peptide receptor radionuclide treatment, chemotherapy, biotherapy)	
Study design	Randomized controlled trials (RCTs)	Case reports
	Prospective and retrospective comparative cohort studies	Editorials
	Noncomparative cohort studies	Reviews
	Case-control studies	
	Case series	
Reporting		Studies that do not report the diagnostic accuracy (first scientific question)
		Studies that do not report the assessment of resectability (second scientific question)
Test of interest	Computed tomography (CT)	
	Magnetic resonance imaging (MRI)	
	Ultrasound scanning	

**Table 6:** Eligibility criteria for review on functional imaging modality.

Study characteristic	Inclusion criteria	Exclusion criteria
Patient population	Patients with NET	Children or adolescents (under the age of 18 years)
	Patients with liver metastases	
Test of interest	SPECT^a^	
	SPECT/CT^b^	
	SRS^c^	
	^123^I-MIBG-Scintigraphy^d^	
	^18^F-FDA-PET^e^	
	^18^F-FDG-PET^f^	
	^18^F-DOPA PET/CT^g^	
	PET/CT^h^	
	PET/MRI^i^	
	^111^In-SRS^j^	
	^123^I-SRS^k^	
Study design	Randomized controlled trials (RCTs)	Case reports
	Prospective and retrospective comparative cohort studies	
	Noncomparative cohort studies	
	Case-control studies	
	Case series	Reviews
Reporting		Studies that do not report the diagnostic accuracy
		Studies that do not report the assessment of resectability

^a^Single photon emission computed tomography

^b^Hybrid method of single photon emission computed tomography and computed tomography

^c^Somatostatin receptor scintigraphy

^d^(123) Iodine-metaiodobenzylguanidine scintigraphy

^e^(18) Fluoro-dopamine positron emission tomography

^f^(18) Fluoro-2-deoxy-D-glucose positron emission tomography

^g^ (18) Fluoro-L-dihydroxyphenylalanine positron emission tomography

^h^Hybrid method of positron emission tomography and computed tomography

^i^ Hybrid method of positron emission tomography and magnetic resonance imaging

^j^(111) Indium-somatostatin receptor scintigraphy

^k^(123) Iodine-somatostatin receptor scintigraphy

**Table 7:** Eligibility criteria for biopsy of primary and liver metastases.

Study characteristic	Inclusion criteria	Exclusion criteria
Patient population	Patients with primary neuroendocrine tumors and/or NET liver metastases	Children or adolescents (under the age of 18 years old)
	Patients who underwent a biopsy of the primary and liver metastasis	
	Patients who underwent multiple biopsies of the liver metastases and/or healthy parenchyma	
Test of interest	Biopsy of primary NET and/or NET liver metastases	Studies that do not report histo-pathological biopsy results
Study design	Randomized controlled trials (RCTs)	Case reports
	Prospective and retrospective comparative cohort studies	
Noncomparative cohort studies	Case-control studies	Reviews
	Case series	
	Cross-sectional and/or cohort studies	

### Search

Librarians of the Medical Library Careum, University of Zurich, Switzerland, develop the electronic search strategy to query databases and to identify all potentially relevant articles. The following databases will be searched: MEDLINE, EMBASE and The Cochrane Library (Cochrane Database of Systematic Reviews, Cochrane Central Register of Controlled Trials (CENTRAL), Database of Abstracts of Reviews of Effects). The investigators will be provided with an Endnote file containing all identified titles and, if available, the corresponding abstracts. Additional articles will be retrieved through manual search or scanning of reference lists. Titles and/or abstracts of all identified records will be independently screened by two review team members to ascertain their relevance and to identify studies that potentially meet the inclusion criteria outlined in Tables 2-5. The full text of each of these potentially relevant studies will then be assessed for eligibility. Any disagreement will be resolved through discussion with a third review team member. A predeﬁned protocol will be used to extract data from the included studies for assessment of study quality and evidence synthesis.

### Data Extraction

The parameters for data extraction will be the following: first author’s name, publication year, answering scientific questions, study design, total number of patients, number of patients in the study group, and number of patients in the comparison group. The Grading of Recommendations Assessment, Development and Evaluation (GRADE) will be used to grade the quality (level) of evidence and the strength of recommendations [35].

A narrative synthesis of the ﬁndings from studies included will be provided. A quantitative synthesis will be used for studies that are sufficiently homogenous from a clinical (comparability of populations, interventions and outcomes) and from a statistical perspective (heterogeneity, eg, I2<50%). We anticipate that there will be a limited scope for meta-analysis despite a relatively large number of studies due to the different outcome measurements of the existing trials (ie, since such tumors are rare). However, results from studies using the same type of intervention and comparator, with the same outcome and measurements will be pooled using a random-effects meta-analysis. In addition risk ratios for binary outcomes, 95% conﬁdence intervals and two- sided P values will be calculated for each outcome.

## Discussion

There are several modalities for the diagnosis and prediction of neuroendocrine liver metastases; however, there is a lack of consensual data on the subject. The six systematic reviews described in this protocol will elucidate the role and compare histopathological prognostic and biochemical markers, biopsies of the primary neuroendocrine tumor and NET liver metastases, morphological and functional imaging modalities. They will help to define clinical guidelines.

## Multimedia Appendix 1

CONSORT-EHEALTH checklist V1.6.2 [36].

## Abbreviations

CT: computed tomography
E-AHPBA: European-African Hepato-Pancreato-Biliary Association
GRADE: Grading of Recommendations Assessment, Development and Evaluation
MRI: magnetic resonance imaging
NET: neuroendocrine tumors
PET: positron emission tomography
RCT: randomized controlled trial
US: ultrasound scanning
WHO: World Health Organization
