# Metadata Correction: Diagnosis and Prediction of Neuroendocrine Liver Metastases: A Protocol of Six Systematic Reviews

**DOI:** 10.2196/resprot.3488

**Published:** 2014-04-28

**Authors:** Stephan Arigoni, Stefan Ignjatovic, Patrizia Sager, Jonas Betschart, Tobias Buerge, Elena Scherrer, Josephine Wachtl, Christoph Tschuor, Perparim Limani, Milo A Puhan, Mickael Lesurtel, Dimitri A Raptis, Stefan Breitenstein

**Affiliations:** ^1^ Clinic for Visceral and Transplantation Surgery Department of Surgery University Hospital Zurich Zurich Switzerland; ^2^ Institute for Social and Preventive Medicine University of Zurich Zurich Switzerland; ^3^ Cantonal Hospital Winterthur Department of Surgery Clinic for Visceral and Thoracic Surgery Winterthur Switzerland

The author Elena Scherrer was inadvertently omitted from the list of authors during the submission process of the paper "Diagnosis and Prediction of Neuroendocrine Liver Metastases: A Protocol of Six Systematic Reviews" (JMIR Res Protoc 2013;2(2):e60). The author Elena Scherrer (Clinic for Visceral and Transplantation Surgery, Department of Surgery, University Hospital Zurich, Zurich, Switzerland) should have been added after Tobias Buerge in the original published manuscript. This error was corrected in the online version of the paper on the JMIR Research Protocols website on April 28, 2014 along with the publication of this correction notice. This correction notice has been sent to PubMed and the correct full-text has been resubmitted to Pubmed Central and other full-text repositories.

