# Supplementary material for: Diagnosis and Prediction of Neuroendocrine Liver Metastases: A Protocol of Six Systematic Reviews
Source: JMIR Res Protoc. 2013 Dec 23;2(2):e60. doi: 10.2196/resprot.2890 (PMC3875889; doi:10.2196/resprot.2890)
Supplement: Supplementary file 1 [file resprot_v2i2e60_app1.pdf]

CONSORT-EHEALTH Checklist V1.6.2 Report

(based on CONSORT-EHEALTH V1.6), available at [http://tinyurl.com/consort-ehealth-v1-6].

2890

Date completed

10/5/2013 10:27:34

by

Stefan Breitenstein

Diagnosis and Prediction of Neuroendocrine Liver Metastases – A Protocol of Six Systematic Reviews

TITLE

1a-i) Identify the mode of delivery in the title

The manuscript submitted is a protocol, therefore not applicable.

1a-ii) Non-web-based components or important co-interventions in title

The manuscript submitted is a protocol, therefore not applicable.

1a-iii) Primary condition or target group in the title

Diagnosis and Prediction of Neuroendocrine Liver Metastases

ABSTRACT

1b-i) Key features/functionalities/components of the intervention and comparator in the METHODS section of the ABSTRACT

The manuscript submitted is a protocol, therefore not applicable.

1b-ii) Level of human involvement in the METHODS section of the ABSTRACT

The manuscript submitted is a protocol, therefore not applicable.

1b-iii) Open vs. closed, web-based (self-assessment) vs. face-to-face assessments in the METHODS section of the ABSTRACT

The manuscript submitted is a protocol, therefore not applicable.

1b-iv) RESULTS section in abstract must contain use data

The manuscript submitted is a protocol, therefore not applicable.

1b-v) CONCLUSIONS/DISCUSSION in abstract for negative trials

The manuscript submitted is a protocol, therefore not applicable.

INTRODUCTION

2a-i) Problem and the type of system/solution

Neuroendocrine tumours
Neuroendocrine tumours (NETs) arise from the diffuse neuroendocrine system and therefore appear widespread over the whole body, especially in the gastrointestinal tract and the bronchopulmonary system [1, 2]. NETs secreting hormones lead to a symptomatic disease. Non-secreting NETs may occur initially asymptomatic or with delayed symptoms due to progressive increase in tumour mass [3, 4]. Therefore, differences in functional behaviour are the basis of a classification system categorising functioning and non-functioning NETs [4]. Other reported classification systems are based on embryological origin or histopathological findings. The World Health Organization (WHO) presented in 2010 a new classification on the basis of tumour grading using histopathological criteria such as Ki 67 index, mitotic count, and presence or absence of necrosis [5].
NETs are a relative rare disease with an incidence 1-3/100'000 [6, 7]. The large range of reported incidence might be due to the fact that NETs often present initially asymptomatic and are often found accidentally or in autopsies [4]. Preponderantly, NETs emerge sporadically (>90%) and are traditionally assigned to multiple endocrine neoplasia type 1 (MEN1), neurofibromatosis-type 1 (NF1), and Von-Hippel-Lindau syndrome [1, 4]. The clinical picture of NETs span over the different effects of excessive hormone secretion such as hypergastrinemia in Zollinger-Ellison Syndrome (ZES) with hyperchlorhydria, hyperinsulinemia in insulinoma, flushing and diarrhoea in the serotoninergic carcinoid syndrome. In the case of non-secreting NETs, symptoms present due to the adverse effects of the growing primary tumour or metastases [8].

Biochemical markers
Hormones secreted from NETs can be used as specific markers for NETs. Moreover, NETs express, store, and secrete characteristic neuronal proteins such as acid glycoprotein chromogranin A (a component of the membrane neurosecretory granula), neuron-specific-enolase (NSE), and synaptophysin [3, 9]. These proteins derived from neuronal structures could serve as markers and are even positive in non-functioning NETs [1, 3]. Since more than one half of NETs are non-secreting, these proteins play a crucial role [4]. Assessment of the different biochemical markers depends on various parameters, such as threshold cut-off level, detecting method of urine, serum or plasma as well as location of the primary tumour or metastases and extension of the disease. In consideration of the great number of evaluation parameters the comparison of studies is difficult [10, 11].

Histopathological prognostic markers
Ki67 is a monoclonal antibody, which was introduced in 1984 by Gerdes et al. [11]. It detects a growth rate depending on the nuclear antigen Ki67 which is only expressed during active cell cycle phases (S, G2 and M-phase). Ki67 is completely absent during the resting phase G0. Therefore, cell proliferation is assessed by the immunohistologic presence of Ki67 positive cells per area in stained tissue blocks [11].
For various human neoplasms such as breast, lung, and solid cancers, Ki67 proliferation index has been successfully established as a predictive marker [12, 13]. The higher the cell proliferation, the greater is the probability for metastases with consecutively decreased patient survival. The primary location of NETs metastases is the liver [14, 15, 16, 17]. The occurrence of hepatic metastases is a prognostic factor which strongly influences the survival of patients suffering from NET [18, 19, 20].

Genetic signatures and the presence of circulating tumour cells
To stratify outcomes in patients undergoing resection of primary NET, a simple scoring system using tumour size, histological grade, nodal metastases, and resection margin status has been introduced [21]. Nevertheless, current classification systems for NETs other than PET fail to predict the clinical course and the response to treatment [22]. The discrepancy might be explained either by an insufficient accuracy of these classification systems or an adaptive NET behaviour [23]. These limitations of the pathologic classifications have led to the investigation of other predictive parameters based on genetic signatures as well as the presence of circulating tumour cells [24, 25]. These novel predictive parameters have to be included in the classification systems in order to take in count the biological behaviour, the likelihood for developing metastases as well as the choice of treatment [25].

Imaging methods
Imaging methods are used to diagnose neuroendocrine tumours (NETs) and their metastases [26]. Beside conventional morphologic imaging methods, functional imaging modalities have been introduced in order to improve accuracy in detecting NETs and liver metastases [27]. Functional imaging methods have their limitations with a great impact on a possible therapeutic strategy, where differentiation between pancreatic foci and neighbouring lymph nodes as well as exact demarcation of a suspicious focus to a liver segment is crucial [28]. Advanced techniques such as contrast-enhanced ultrasound may assist in earlier detection of hepatic metastases, and could therefore offer a wider therapeutic range either surgically, with radiofrequency thermal ablation or with systemic chemotherapy [29].

Liver biopsy
The most common site of neuroendocrine tumour (NET) metastases is the liver [30]. The presence of hepatic metastases is a strong prognostic factor for the survival of patients with NETs, regardless of the primary tumour site [31]. Histologic examination is the most sensitive diagnostic method and forms the basis for treatment decisions [32]. However, the value of the biopsy for treatment decision making involving primary NETs and their liver metastases is not well defined [33, 34].

The aim of these six systematic reviews is to assess the predictive value of Ki67 index and other biomarkers, to compare the diagnostic accuracy of morphological and functional imaging, and to define the role of biopsy in the diagnosis and prediction of neuroendocrine tumour liver metastases.

2a-ii) Scientific background, rationale: What is known about the (type of) system

Neuroendocrine tumours
Neuroendocrine tumours (NETs) arise from the diffuse neuroendocrine system and therefore appear widespread over the whole body, especially in the gastrointestinal tract and the bronchopulmonary system [1, 2]. NETs secreting hormones lead to a symptomatic disease. Non-secreting NETs may occur initially asymptomatic or with delayed symptoms due to progressive increase in tumour mass [3, 4]. Therefore, differences in functional behaviour are the basis of a classification system categorising functioning and non-functioning NETs [4]. Other reported classification systems are based on embryological origin or histopathological findings. The World Health Organization (WHO) presented in 2010 a new classification on the basis of tumour grading using histopathological criteria such as Ki 67 index, mitotic count, and presence or absence of necrosis [5].
NETs are a relative rare disease with an incidence 1-3/100'000 [6, 7]. The large range of reported incidence might be due to the fact that NETs often present initially asymptomatic and are often found accidentally or in autopsies [4]. Preponderantly, NETs emerge sporadically (>90%) and are traditionally assigned to multiple endocrine neoplasia type 1 (MEN1), neurofibromatosis-type 1 (NF1), and Von-Hippel-Lindau syndrome [1, 4]. The clinical picture of NETs span over the different effects of excessive hormone secretion such as hypergastrinemia in Zollinger-Ellison Syndrome (ZES) with hyperchlorhydria, hyperinsulinemia in insulinoma, flushing and diarrhoea in the serotoninergic carcinoid syndrome. In the case of non-secreting NETs, symptoms present due to the adverse effects of the growing primary tumour or metastases [8].

Biochemical markers
Hormones secreted from NETs can be used as specific markers for NETs. Moreover, NETs express, store, and secrete characteristic neuronal proteins such as acid glycoprotein chromogranin A (a component of the membrane neurosecretory granula), neuron-specific-enolase (NSE), and synaptophysin [3, 9]. These proteins derived from neuronal structures could serve as markers and are even positive in non-functioning NETs [1, 3]. Since more than one half of NETs are non-secreting, these proteins play a crucial role [4]. Assessment of the different biochemical markers depends on various parameters, such as threshold cut-off level, detecting method of urine, serum or plasma as well as location of the primary tumour or metastases and extension of the disease. In consideration of the great number of evaluation parameters the comparison of studies is difficult [10, 11].

Histopathological prognostic markers
Ki67 is a monoclonal antibody, which was introduced in 1984 by Gerdes et al. [11]. It detects a growth rate depending on the nuclear antigen Ki67 which is only expressed during active cell cycle phases (S, G2 and M-phase). Ki67 is completely absent during the resting phase G0. Therefore, cell proliferation is assessed by the immunohistologic presence of Ki67 positive cells per area in stained tissue blocks [11].
For various human neoplasms such as breast, lung, and solid cancers, Ki67 proliferation index has been successfully established as a predictive marker [12, 13]. The higher the cell proliferation, the greater is the probability for metastases with consecutively decreased patient survival. The primary location of NETs metastases is the liver [14, 15, 16, 17]. The occurrence of hepatic metastases is a prognostic factor which strongly influences the survival of patients suffering from NET [18, 19, 20].

Genetic signatures and the presence of circulating tumour cells
To stratify outcomes in patients undergoing resection of primary NET, a simple scoring system using tumour size, histological grade, nodal metastases, and resection margin status has been introduced [21]. Nevertheless, current classification systems for NETs other than PET fail to predict the clinical course and the response to treatment [22]. The discrepancy might be explained either by an insufficient accuracy of these classification systems or an adaptive NET behaviour [23]. These limitations of the pathologic classifications have led to the investigation of other predictive parameters based on genetic signatures as well as the presence of circulating tumour cells [24, 25]. These novel predictive parameters have to be included in the classification systems in order to take in count the biological behaviour, the likelihood for developing metastases as well as the choice of treatment [25].

Imaging methods
Imaging methods are used to diagnose neuroendocrine tumours (NETs) and their metastases [26]. Beside conventional morphologic imaging methods, functional imaging modalities have been introduced in order to improve accuracy in detecting NETs and liver metastases [27]. Functional imaging methods have their limitations with a great impact on a possible therapeutic strategy, where differentiation between pancreatic foci and neighbouring lymph nodes as well as exact demarcation of a suspicious focus to a liver segment is crucial [28]. Advanced techniques such as contrast-enhanced ultrasound may assist in earlier detection of hepatic metastases, and could therefore offer a wider therapeutic range either surgically, with radiofrequency thermal ablation or with systemic chemotherapy [29].

Liver biopsy
The most common site of neuroendocrine tumour (NET) metastases is the liver [30]. The presence of hepatic metastases is a strong prognostic factor for the survival of patients with NETs, regardless of the primary tumour site [31]. Histologic examination is the most sensitive diagnostic method and forms the basis for treatment decisions [32]. However, the value of the biopsy for treatment decision making involving primary NETs and their liver metastases is not well defined [33, 34].

The aim of these six systematic reviews is to assess the predictive value of Ki67 index and other biomarkers, to compare the diagnostic accuracy of morphological and functional imaging, and to define the role of biopsy in the diagnosis and prediction of neuroendocrine tumour liver metastases.

METHODS

3a) CONSORT: Description of trial design (such as parallel, factorial) including allocation ratio
The manuscript submitted is a protocol, therefore not applicable.
3b) CONSORT: Important changes to methods after trial commencement (such as eligibility criteria), with reasons
The manuscript submitted is a protocol, therefore not applicable.
3b-i) Bug fixes, Downtimes, Content Changes
The manuscript submitted is a protocol, therefore not applicable.
4a) CONSORT: Eligibility criteria for participants
The manuscript submitted is a protocol, therefore not applicable.There were no restrictions in the literature search regarding the publication language or by publication date. The following study types were included: Randomised Controlled Trials (RCTs), prospective and retrospective comparative cohort and case-control studies and case series
4a-i) Computer / Internet literacy
The manuscript submitted is a protocol, therefore not applicable.
4a-ii) Open vs. closed, web-based vs. face-to-face assessments:
The manuscript submitted is a protocol, therefore not applicable.
4a-iii) Information giving during recruitment
The manuscript submitted is a protocol, therefore not applicable.
4b) CONSORT: Settings and locations where the data were collected

The manuscript submitted is a protocol, therefore not applicable.
4b-i) Report if outcomes were (self-)assessed through online questionnaires
The manuscript submitted is a protocol, therefore not applicable.
4b-ii) Report how institutional affiliations are displayed
The manuscript submitted is a protocol, therefore not applicable.
5) CONSORT: Describe the interventions for each group with sufficient details to allow replication, including how and when they were actually administered
5-i) Mention names, credential, affiliations of the developers, sponsors, and owners
The manuscript submitted is a protocol, therefore not applicable.
5-ii) Describe the history/development process
The manuscript submitted is a protocol, therefore not applicable.
5-iii) Revisions and updating
The manuscript submitted is a protocol, therefore not applicable.
5-iv) Quality assurance methods
The manuscript submitted is a protocol, therefore not applicable.
5-v) Ensure replicability by publishing the source code, and/or providing screenshots/screen-capture video, and/or providing flowcharts of the algorithms used
The manuscript submitted is a protocol, therefore not applicable.
5-vi) Digital preservation
The manuscript submitted is a protocol, therefore not applicable.
5-vii) Access
The manuscript submitted is a protocol, therefore not applicable.
5-viii) Mode of delivery, features/functionalities/components of the intervention and comparator, and the theoretical framework
The manuscript submitted is a protocol, therefore not applicable.
5-ix) Describe use parameters
The manuscript submitted is a protocol, therefore not applicable.
5-x) Clarify the level of human involvement
The manuscript submitted is a protocol, therefore not applicable.
5-xi) Report any prompts/reminders used
The manuscript submitted is a protocol, therefore not applicable.
5-xii) Describe any co-interventions (incl. training/support)
The manuscript submitted is a protocol, therefore not applicable.
6a) CONSORT: Completely defined pre-specified primary and secondary outcome measures, including how and when they were assessed
The manuscript submitted is a protocol, therefore not applicable.
6a-i) Online questionnaires: describe if they were validated for online use and apply CHERRIES items to describe how the questionnaires were designed/deployed
The manuscript submitted is a protocol, therefore not applicable.
6a-ii) Describe whether and how "use" (including intensity of use/dosage) was defined/measured/monitored
The manuscript submitted is a protocol, therefore not applicable.
6a-iii) Describe whether, how, and when qualitative feedback from participants was obtained
The manuscript submitted is a protocol, therefore not applicable.
6b) CONSORT: Any changes to trial outcomes after the trial commenced, with reasons
The manuscript submitted is a protocol, therefore not applicable.
7a) CONSORT: How sample size was determined
7a-i) Describe whether and how expected attrition was taken into account when calculating the sample size
The manuscript submitted is a protocol, therefore not applicable.
7b) CONSORT: When applicable, explanation of any interim analyses and stopping guidelines
The manuscript submitted is a protocol, therefore not applicable.
8a) CONSORT: Method used to generate the random allocation sequence
The manuscript submitted is a protocol, therefore not applicable.
8b) CONSORT: Type of randomisation; details of any restriction (such as blocking and block size)
The manuscript submitted is a protocol, therefore not applicable.
9) CONSORT: Mechanism used to implement the random allocation sequence (such as sequentially numbered containers), describing any steps taken to conceal the sequence until interventions were assigned
The manuscript submitted is a protocol, therefore not applicable.
10) CONSORT: Who generated the random allocation sequence, who enrolled participants, and who assigned participants to interventions
The manuscript submitted is a protocol, therefore not applicable.
11a) CONSORT: Blinding - If done, who was blinded after assignment to interventions (for example, participants, care providers, those assessing outcomes) and how
11a-i) Specify who was blinded, and who wasn't
The manuscript submitted is a protocol, therefore not applicable.
11a-ii) Discuss e.g., whether participants knew which intervention was the "intervention of interest" and which one was the "comparator"
The manuscript submitted is a protocol, therefore not applicable.
11b) CONSORT: If relevant, description of the similarity of interventions
The manuscript submitted is a protocol, therefore not applicable.
12a) CONSORT: Statistical methods used to compare groups for primary and secondary outcomes
The manuscript submitted is a protocol, therefore not applicable.
12a-i) Imputation techniques to deal with attrition / missing values
The manuscript submitted is a protocol, therefore not applicable.
12b) CONSORT: Methods for additional analyses, such as subgroup analyses and adjusted analyses
The manuscript submitted is a protocol, therefore not applicable.

RESULTS

13a) CONSORT: For each group, the numbers of participants who were randomly assigned, received intended treatment, and were analysed for the primary outcome

The manuscript submitted is a protocol, therefore not applicable.

13b) CONSORT: For each group, losses and exclusions after randomisation, together with reasons

The manuscript submitted is a protocol, therefore not applicable.

13b-i) Attrition diagram

The manuscript submitted is a protocol, therefore not applicable.

14a) CONSORT: Dates defining the periods of recruitment and follow-up

The manuscript submitted is a protocol, therefore not applicable.

14a-i) Indicate if critical "secular events" fell into the study period

The manuscript submitted is a protocol, therefore not applicable.

14b) CONSORT: Why the trial ended or was stopped (early)

The manuscript submitted is a protocol, therefore not applicable.

15) CONSORT: A table showing baseline demographic and clinical characteristics for each group

The manuscript submitted is a protocol, therefore not applicable.

15-i) Report demographics associated with digital divide issues

The manuscript submitted is a protocol, therefore not applicable.

16a) CONSORT: For each group, number of participants (denominator) included in each analysis and whether the analysis was by original assigned groups

16-i) Report multiple "denominators" and provide definitions

The manuscript submitted is a protocol, therefore not applicable.

16-ii) Primary analysis should be intent-to-treat

The manuscript submitted is a protocol, therefore not applicable.

17a) CONSORT: For each primary and secondary outcome, results for each group, and the estimated effect size and its precision (such as 95% confidence interval)

The manuscript submitted is a protocol, therefore not applicable.

17a-i) Presentation of process outcomes such as metrics of use and intensity of use

The manuscript submitted is a protocol, therefore not applicable.

17b) CONSORT: For binary outcomes, presentation of both absolute and relative effect sizes is recommended

The manuscript submitted is a protocol, therefore not applicable.

18) CONSORT: Results of any other analyses performed, including subgroup analyses and adjusted analyses, distinguishing pre-specified from exploratory

The manuscript submitted is a protocol, therefore not applicable.

18-i) Subgroup analysis of comparing only users

The manuscript submitted is a protocol, therefore not applicable.

19) CONSORT: All important harms or unintended effects in each group

The manuscript submitted is a protocol, therefore not applicable.

19-i) Include privacy breaches, technical problems

The manuscript submitted is a protocol, therefore not applicable.

19-ii) Include qualitative feedback from participants or observations from staff/researchers

The manuscript submitted is a protocol, therefore not applicable.

DISCUSSION

20) CONSORT: Trial limitations, addressing sources of potential bias, imprecision, multiplicity of analyses

20-i) Typical limitations in ehealth trials

The manuscript submitted is a protocol, therefore not applicable.There are several modalities for the diagnosis and prediction of neuroendocrine liver metastases, but there is a lack of consensual data on the subject. These six systematic reviews described in this protocol will elucidate the role and compare histopathological prognostic and biochemical markers, biopsies of the primary neuroendocrine tumour and NET liver metastases, morphological and functional imaging modalities. They will help to define clinical guidelines.

21) CONSORT: Generalisability (external validity, applicability) of the trial findings

21-i) Generalizability to other populations

The manuscript submitted is a protocol, therefore not applicable.

21-ii) Discuss if there were elements in the RCT that would be different in a routine application setting

The manuscript submitted is a protocol, therefore not applicable.

22) CONSORT: Interpretation consistent with results, balancing benefits and harms, and considering other relevant evidence

22-i) Restate study questions and summarize the answers suggested by the data, starting with primary outcomes and process outcomes (use)

The manuscript submitted is a protocol, therefore not applicable.

22-ii) Highlight unanswered new questions, suggest future research

The manuscript submitted is a protocol, therefore not applicable.

Other information

23) CONSORT: Registration number and name of trial registry

International Prospective Register of Systematic Reviews (PROSPERO): CRD42012002644, CRD42012002647, CRD42012002651, CRD42012002649, CRD42012002650, CRD42012002651.

24) CONSORT: Where the full trial protocol can be accessed, if available

International Prospective Register of Systematic Reviews (PROSPERO): CRD42012002644, CRD42012002647, CRD42012002651, CRD42012002649, CRD42012002650, CRD42012002651.

25) CONSORT: Sources of funding and other support (such as supply of drugs), role of funders

NO funding.

X26-i) Comment on ethics committee approval

The manuscript submitted is a protocol, therefore not applicable.

x26-ii) Outline informed consent procedures

The manuscript submitted is a protocol, therefore not applicable.

X26-iii) Safety and security procedures

The manuscript submitted is a protocol, therefore not applicable.

X27-i) State the relation of the study team towards the system being evaluated

All authors have reported no potential conflicts of interest existing with any organisations concerning issues discussed in this article.
